# Molecular survey of *Hepatozoon* spp., piroplasmids, and onchocercids in wild birds from the Brazilian Pantanal

**DOI:** 10.1590/S1984-29612025065

**Published:** 2025-11-17

**Authors:** Amir Salvador Alabí Córdova, Ana Cláudia Calchi, Alan Fecchio, Lizeth Fernanda Banguero-Micolta, Rosangela Zacarias Machado, Marcos Rogério André

**Affiliations:** 1 Universidade Estadual Paulista – UNESP, Faculdade de Ciências Agrárias e Veterinárias – FCAV, Departamento de Patologia, Reprodução e Saúde Única, – VBBL, Jaboticabal, SP, Brasil; 2 Academy of Natural Sciences of Drexel University, Department of Ornithology, Philadelphia, PA, USA

**Keywords:** Filarioid, Piroplasm, Hepatozoidae, wetland, avian, Filarioide, Piroplasmas, Hepatozoidae, planície inundável, aves

## Abstract

The diversity of *Hepatozoon* spp., piroplasmids, and onchocercids that parasitize birds worldwide has been underestimated, especially in Brazil. The present work aimed to investigate, using molecular assays, the occurrence of *Hepatozoon* spp., piroplasmids, and onchocercids in tropical birds from the Brazilian Pantanal wetland, in the states of Mato Grosso and Mato Grosso do Sul. Blood sampling and DNA extraction were performed on 517 birds from 13 avian orders. DNA samples positive to endogenous gene (avian *β-actin*) were subjected to PCR assays targeting the 18S rRNA gene of *Hepatozoon* spp. and piroplasmids as well as PCR assays for onchocercids (*cox-1,* 28S rRNA and 18S rRNA genes). As a result, two onchocercids (0.4%) were identified. None was positive in the PCR assays for *Hepatozoon* spp. or piroplasmids. The *cox-1* sequence detected in *Ramphocelus carbo* grouped with *Cardiofilaria* sp., and onchocercid *cox-1* sequence obtained from *Taraba major* grouped with *Splendidofilaria* spp. This is the first molecular report of onchocercids closely related to *Cardiofilaria* spp. and *Splendidofilaria* spp. in birds from the Brazilian Pantanal.

## Resumo

A diversidade de *Hepatozoon* spp., piroplasmídeos e oncocercídeos que parasitam aves em todo o mundo tem sido subestimada, especialmente no Brasil. O presente trabalho teve como objetivo investigar, por meio de ensaios moleculares, a ocorrência de *Hepatozoon* spp., piroplasmídeos e oncocercídeos em aves tropicais do Pantanal brasileiro, nos estados de Mato Grosso e Mato Grosso do Sul. Extrações de DNA foram realizadas em amostras de sangue de 517 aves de 13 ordens. Amostras de DNA positivas para o gene endógeno (*β-actina* aviária) foram submetidas a ensaios de PCR direcionados ao gene 18S rRNA de *Hepatozoon* spp. e piroplasmídeos e ensaios de PCR para oncocercideos (genes *cox-1*, 28S rRNA e 18S rRNA). Como resultado, dois oncocercideos (0,4%) foram identificados. Nenhuma ave foi positiva nos ensaios de PCR para *Hepatozoon* spp. ou piroplasmídeos. A sequência *cox-1* detectada em *Ramphocelus carbo* agrupou-se com *Cardiofilaria* sp., e a sequência de *cox-1* obtida de *Taraba major* agrupou-se com *Splendidofilaria* spp. Este é o primeiro relato molecular de oncocercídeos intimamente relacionados a *Cardiofilaria* spp. e *Splendidofilaria* spp. em aves do Pantanal brasileiro.

## Introduction

The ability of birds to adapt to several ecological niches aids in the spread of vector-borne agents, viruses, ticks, and zoonotic pathogens through avian migration (Schmitt & Edwards, 2022).

*Hepatozoon* spp. (Adeleorina: Hepatozoidae) comprise apicomplexan chromists whose life cycle includes arthropods (ticks, fleas, flies, mosquitoes or lice) as definitive hosts and vertebrates (amphibians, reptiles, mammals, and birds) as intermediate hosts (Lainson et al., 2003). To date, only *Hepatozoon peircei* has been detected in storm petrel (*Oceanodroma melania*) blood samples from Baja California, Mexico, when using a PCR assay targeting the18S rRNA gene (Merino et al., 2014).

Sixteen *Babesia* species have been found in birds (Ebani & Mancianti, 2022). In Brazil, *Babesia vogeli*-like sequences were identified in Orinoco geese (*Neochen jubata*) (Werther et al., 2017), *Babesia poelea* was detected in brown (*Sula leucogaster*) and masked boobies (*Sula dactylatra*) (Quillfeldt et al., 2014), and a unique *Babesia* sequence was found in an albatross (*Thalassarche chlororhynchos*), closely related to strains from Chile and New Zealand (Sgarioni et al., 2023).

The diversity of onchocercid parasites of birds is in fact unknown (Binkienė et al., 2021). Avian onchocercids are mainly transmitted by Nematocera dipterans or lice (order Phthiraptera) (Bartlett, 2008). Avian-associated onchocercids (e.g., *Pelecitus* spp.) may have implications in Public Health due to their ability to parasitize avian and non-avian hosts including humans (Bain et al., 2011; Bartlett, 2008).

The knowledge on the genetic diversity of avian onchocercids is limited. *Chandlerella quiscali* and *Splendidofilaria* spp. 18S rRNA sequences were detected in passerines (*Turdus migratorius* and *Passer domesticus*) from the USA (Hamer et al., 2012). *Filaroidea* spp. 12S rRNA sequences were detected in ramphastid birds (*Ramphastos sulfuratus* and *Aulacorhyncus prasimus*) from Mexico (Sanchez-Godoy et al., 2020). In Lithuania, *Eufilaria acrocephalusi*, *Eufilaria sylviae* and *Splendidofilaria bartletti* were detected in passerines (*Acrocephalus arundinaceus*, *Sylvia atricapilla*, *Sylvia borin* and *Sylvia curruca*) when targeting the 28S rRNA and *cox-1* genes (Binkienė et al., 2021). Recently, in the Pantanal of Mato Grosso state, Onchocercid *cox-1* sequences related to *Aproctella* spp. were detected in *Ramphocelus carbo*, *Turdus amaurocalinus,* and *Synallaxis albilora*, whereas one sequence detected in *R. carbo* was ancestral to the clade comprising *Splendidofilaria* spp. and *Eufilaria* spp. (Alabí Córdova et al., 2025).

The present study aimed to investigate, using molecular techniques, the ocurrence of *Hepatozoon* spp., piroplasmids, and onchocercids in wild birds sampled from the Brazilian Pantanal wetland, the largest floodplain in South America.

## Material and Methods

### Study area and sampling

Between April and August to November 2019, twenty mist nets (36 mm mesh, 12 m length, 2.5 m height) were deployed along trails across four sites within the Pantanal region: Nossa Senhora do Livramento (99 samples), Poconé (100 samples), and Santo Antonio de Leverger (200 samples) in Mato Grosso, as well as Corumbá (101 samples) in Mato Grosso do Sul. Sampling at each locality was conducted over five days following the protocol described by Alabí Córdova et al. (2024a).

Bird identification was carried out by an experienced ornithologist using field guides, and blood samples were collected with proper permits in accordance with Brazilian regulations (IBAMA 72548 & 72790), the FCAV/UNESP Ethics Committee (CEUA 268/21), and SISGEN (AF30FD1) (Alabí Córdova et al., 2024a). A total of 517 birds from 13 orders were sampled (see Supplementary Material Table SM1).

### DNA extraction and PCR for endogenous gene

DNA extraction and a conventional PCR assay targeting the avian *β-actin* gene were previously performed, mean concentrations and 260/280 ratios of avian blood DNA samples were detailed in previous studies (Alabí Córdova et al., 2024a, b). 

### Molecular screening and molecular characterization for *Hepatozoon* spp., piroplasmids and onchocercids

DNA samples positive for avian *β-actin* (Supplementary Material Table SM1) were subjected to screening PCR assays targeting the 18S rRNA gene of *Hepatozoon* spp. and piroplasmids and *cox-1* gene for onchocercids (Supplementary Material Table SM2). Each reaction had a total volume of 25 µL, containing 1.25 U Go Taq Hot Start Polymerase (Promega®, Madison, WI, USA), PCR buffer 10× (Promega®, Madison, WI, USA), sterilized ultra-pure water (Invitrogen®, Carlsbad, CA, USA), 0.2 mM of each deoxynucleotide, 0.4 µM of each oligonucleotide, 3.0 mM of MgCl_2_, and 3 µL DNA template. In nested PCR assays, 1 μL of the amplified product from the first PCR was used as the DNA template in the second reaction. DNA samples from *Hepatozoon canis* (Calchi et al., 2024), *Babesia vogeli* (Jaboticabal strain) (Furuta et al., 2009), and *Dirofilaria immitis* (obtained from female adult worms which were kindly provided by Dr. Norma Labarthe, Oswaldo Cruz Foundation - FIOCRUZ, Rio de Janeiro) were used as positive controls. Ultra-pure sterilized water was used as a negative control. Avian blood DNA samples that were positive in the PCR assay *cox-1* gene for onchocercids were subjected to conventional PCR assays targeting the 18S rRNA and 28S rRNA genes (Supplementary Material Table SM2).

### Sequencing and BLASTn analysis

Amplicons were purified using the Wizard SV Gel and PCR cleanup System kit (Promega, Madison, WI), in accordance with the manufacturers’ recommendations.

The purified PCR products were subjected to Sanger sequencing in an ABI PRISM 3700 DNA Analyzer (Applied Biosystems, Waltham, MA, USA) at “Centro de Recursos Biológicos e Biologia Genômica” (CREBIO-FCAV-UNESP, Jaboticabal, SP, Brazil). Sequences were assembled using BioEdit v7.2.5 software and compared with homologous sequences deposited in GenBank (Benson et al., 2004) (accessed on October 12, 2024) using BLASTn.

### Phylogenetic analyses

Sequence trimming, consensus sequences, alignment with homologous sequences downloaded from GenBank and phylogenetic inference was previously described by Alabí Córdova et al. (2024b). Bootstrap values for the maximum likelihood (ML) were analyzed with 100 repetitions using IQtree online version (accessed on October 10, 2024) (Felsenstein, 1985). The phylogenetic tree was edited using Figtree V1.4.4 software (Rambaut, 2018).

## Results

Of the 517 bird blood DNA samples tested for the avian *β-actin* gene by PCR, 500 were positive (Alabí Córdova et al., 2024a).

**Figure 1:**
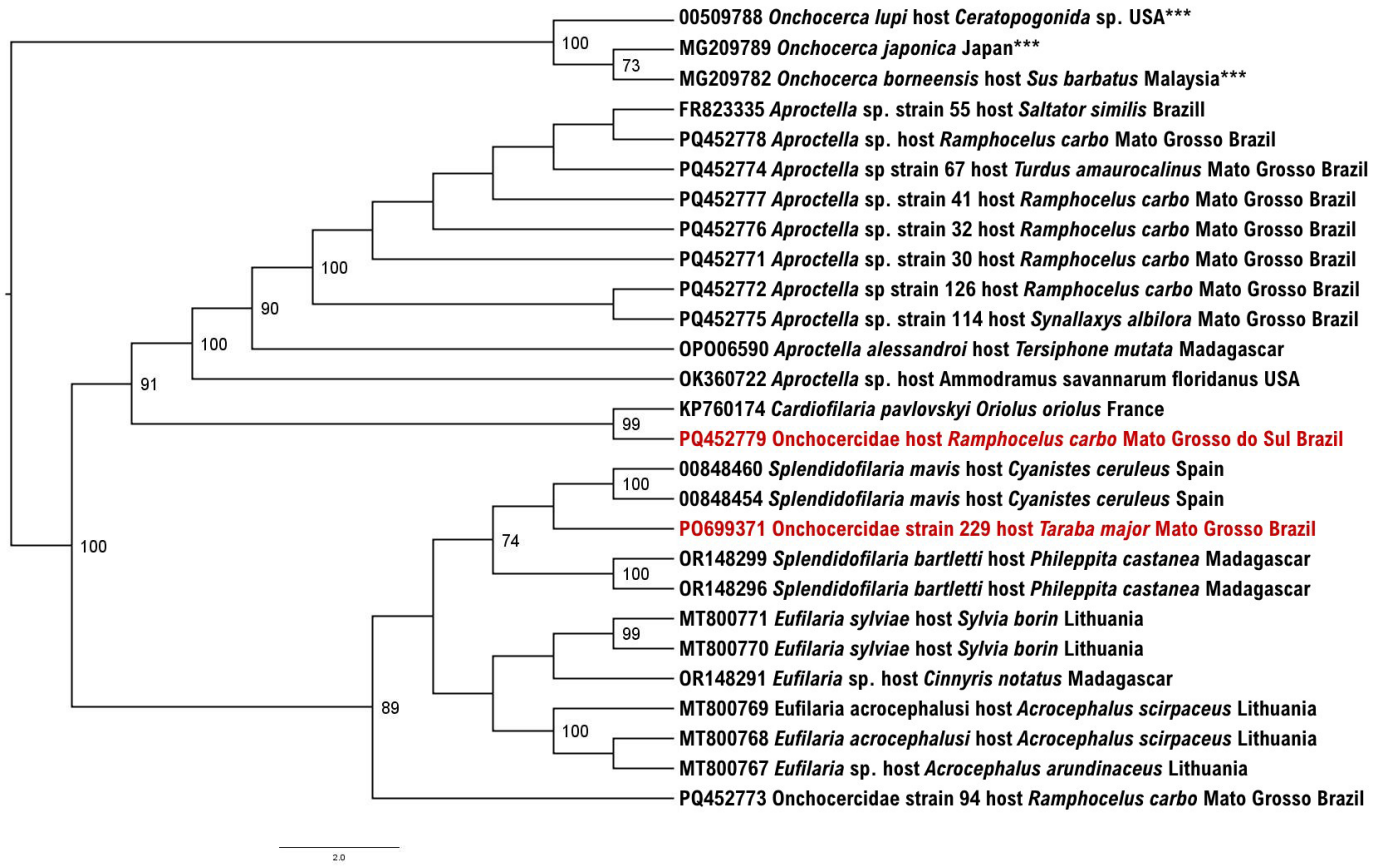
Phylogenetic analysis inferred by maximum likelihood method (GTR+F+I+G4 evolutionary model) based on a 660 bp alignment of the *cox*1 gene for onchocercids, containing 27 onchocercid homologous sequences (*Aproctella*, *Eufilaria*, *Chandlerella*, *Cardiofilaria* and *Splendidofilaria*). *Onchocerca* species (MG209782, MG209789 and OQ509788) were used as outgroups. Sequences obtained in present study are highlighted in red and with (***). Only the bootstraps with values of 70 or more are shown.

In the PCR assay for onchocercids based on the *cox-1* gene, two samples (0.4%) were positive: 229 (BAP114) *Taraba major* from Poconé, Mato Grosso State, and 179 (BEP415) *R. carbo* from Corumbá, Mato Grosso do Sul State. The sequence obtained from *T. major* 229 (BAP114) showed 91% identity (query cover = 99%; E-value = 0.0) with the Onchocercidae (OR148297) obtained from *Neodrepanis coruscans* from Madagascar. The sequence obtained from *R. carbo* 179 (BEP415) showed 92.3% identity (query cover = 99%; E-value = 0.0) with that of *Cardiofilaria pavlolvskyi* (KP760174) obtained from a European golden oriole (*Oriolus oriolus*). In the phylogenetic analysis performed using the Maximum Likelihood method, GTR+F+I+G4 evolutionary model and based on an alignment of 660 bp of the *cox-1* gene, the obtained sequences were positioned into two different clades along with other avian-associated onchocercids. The sequence detected in *R. carbo* (PQ452779) grouped with a *Cardiofilaria* spp. detected in France supported by 99% bootstrap, whereas the sequence obtained from a *T. major* (PQ699371) was closely related to *Splendidofilaria* spp. detected in Spain (OQ848460 and OQ848454) and Madagascar (OR148299 and OR148296), supported by 74% bootstrap (Figure 1). All samples positive to onchocercid *cox-1* based PCR were negative in the additional molecular assays based on the 18S rRNA and 28S rRNA genes.

None of the samples were positive in the PCR assays for *Hepatozoon* spp. or piroplasmids based on the 18S rRNA gene. 

## Discussion

Previous studies carried out in Brazil, based on morphological analysis, reported the occurrence of onchocercids in Parulidae, Picidae, Thamnophilidae and Pipridae birds in the Cerrado biome (Silveira et al., 2010; Ribeiro et al., 2020), in Strigidae in Cerrado – Atlantic Forest interphase (Silva et al., 2014), and in Thamnophilidae, Tyrannidae, Fringillidae birds in Atlantic Forest biome (Bain et al., 1981; Lefoulon et al., 2015; Sebaio et al., 2012). Recently, Alabí Córdova et al. (2025) detected onchocercids of the genus *Aproctella* spp. in Furnariidae, Thraupidae, Turdidae birds and an onchocercid related to *Eufilaria* spp. and *Splendidofilaria* spp. in a Thraupidae bird from the Brazilian Pantanal. In the present study, a molecular occurrence of 0.4% was reported among birds from Thraupidae and Thamnophilidae families, sampled in the Pantanal wetlands, in the states of Mato Grosso and Mato Grosso do Sul. Based on microscopy and/or molecular assays, previous studies reported an overall occurrence of onchocercids in birds ranging from 0.4 to 13.8% (Bartlett, 1992; Silveira et al., 2010; Haas et al., 2011; Sebaio et al., 2012; Silva et al., 2014; Ribeiro et al., 2020). The low molecular occurrence of onchocercids found in the present study when compared to previous studies could be explained by geographical differences, avian host species susceptibility, seasonality, or limitations of the specific *cox-1* gene target for broader filarioid detection.

The phylogenetic analyses based on the *cox-1* gene for avian-associated onchocercids showed herein displayed a similar topology as those previously presented by Hayashi et al. (2024) and Alabí Córdova et al. (2025) based on the same molecular marker. Additionally, the topology presented in this study corroborate with phylogenetic analyses based on a concatenated dataset of several genes (12S rDNA, *cox-1*, *rbp1, hsp70, myoHC*, 18S rDNA, and 28S rDNA) (Lefoulon et al., 2015). The *cox-1* sequence detected in *R. carbo* grouped with *Cardiofilaria* sp.. Altogether, *Cardiofilaria* spp., and *Aproctella* spp. formed a large clade, as previously described (Lefoulon et al., 2015). The Onchocercidae *cox-1* sequence detected in *T. major* was closely related to *Splendidofilaria* spp. detected in Spain (Garrido-Bautista et al., 2023).

The phylogenetic analysis presented here shows that *Splendidofilaria* spp. are closely related to *Eufilaria* spp., and *Cardiofilaria* spp. are closely related to *Aproctella* spp., as previously described in earlier studies (Binkienė et al., 2021; Garrido-Bautista et al., 2023; Hayashi et al., 2024).

Future studies aiming to add more avian-associated *cox-1* onchocercid sequences in the GenBank database are needed in order to shed some light on the phylogenetic diversity of onchocercids among neotropical birds. A noteworthy limitation of this study was the lack of blood smears that might have allowed the description of morphological features of microfilariae and the association with the molecular identity of the detected onchocercids.

Unlike earlier research that found *Hepatozoon peircei* in storm petrels (*Oceanodroma melanaria*) (Merino et al., 2014), this study detected no *Hepatozoon* spp. in birds from Pantanal. The absence of PCR positivity might be due to low parasitemia presented by the sampled birds or the absence of competent vectors in the studied region. In addition, the limited availability of avian-associated *Hepatozoon* 18S rRNA sequences in GenBank hampered designing primers specific for *Hepatozoon* species that infect birds. Nonetheless, the primers used herein have been used to detect a wide diversity of *Hepatozoon* spp. in wild and domestic animals (Thomas et al., 2024). The development of primers specific to avian-related *Hepatozoon* species or targeting alternative molecular markers in the future may improve detection, ensuring accurate detection and identification of this group of protozoa that has been scarcely studied among birds.

Piroplasmida DNA was not detected in the analyzed bird blood samples. It is well known that the occurrence of these agents in birds is low (Ebani & Mancianti, 2022). Indeed, among the few studies conducted in Brazil, the reported positivity rate ranging from 9.7% to 14.3%, with positive birds belonging to the following species: *S. leucogaster*, *S. dactylatra*, and *N. jubata* (Quillfeldt et al., 2014; Werther et al., 2017). Similar to the results found for *Hepatozoon* spp., the lack of Piroplasmida detection in birds sampled in the present study might be related to the low parasitemia at the time of blood sampling or the lack of competent vectors in the Brazilian Pantanal. Although this study used the nested PCR technique, which is more sensitive than conventional PCR, Piroplasmida DNA was not detected in these samples. It is likely that the use of more sensitive techniques, such as digital PCR, may be more effective (Calchi et al., 2025). Furthermore, primers commonly used to detect piroplasmids, which are very effective in mammals, are less efficient in birds, often due to their annealing to the host's DNA, which further complicates reliable detection. Nonetheless, the primers used herein have been shown to be able to detect a wide diversity of piroplasmids, allowing indeed the detection of piroplasms belonging to the 15 Piroplasmida phylogenetic clades described so far (Anjos Pacheco et al., 2025).

## Conclusion

Onchocercids related to *Cardiofilaria* spp. and *Splendidofilaria* spp. were, for the first time, molecularly detected in Brazilian birds. The lack of positivity in the molecular assays for piroplasmids or *Hepatozoon* spp. might be due to the low parasitemia at the time of avian blood sampling, the lack of competent vectors in the studied region, or the inability of the primers used herein in annealing to avian-associated *Hepatozoon*/Piroplasmida species. The use of more sensitive molecular techniques (e.g., digital PCR) and design of primers specific to avian-associated *Hepatozoon*/Piroplasmida in the future are much needed in order to unravel the diversity of these vector-borne protozoa in wild birds from Brazil. Last but not least, the morphological description of microfilariae and adult onchocercids parasitizing birds in Brazil, together with molecular characterization, will provide a deeper understanding of the diversity of this group of filarioids in birds from South America.

## Supplementary Material

Supplementary material accompanies this paper.

Supplementary Table SM1 Sampled birds in the Pantanal wetland in the localities of Nossa Senhora do Livramento (NS), Poconé (Po), and Santo Antonio de Leverger (SA) in the state of Mato Grosso (MT), and Corumbá (Co) in the state Mato Grosso do Sul (MS).

Supplementary Material Table SM2 Description of primers and target genes used in the on conventional PCR assays to detect piroplasmids, Hepatozoon spp., and filariids in avian samples from the Brazilian Pantanal.

This material is available as part of the online article from https://doi.org/10.1590/S1984-29612025065
