# Supplementary material for: Molecular survey of Hepatozoon spp., piroplasmids, and onchocercids in wild birds from the Brazilian Pantanal
Source: Rev Bras Parasitol Vet. 2025 Nov 17;34(4):e010725. doi: 10.1590/S1984-29612025065 (PMC12643243; doi:10.1590/S1984-29612025065)
Supplement: Supplementary Table SM1 [file rbpv-34-4-e010725-suppl01.pdf]

Supplementary Table SM1. Sampled birds in the Pantanal wetland in the localities of Nossa Senhora do Livramento (NS), Poconé (Po), and Santo Antonio de Leverger (SA) in the state of Mato Grosso (MT), and Corumbá (Co) in the state Mato Grosso do Sul (MS).

Sample number	Species	Common name	Locality	State	PCR for *avian β-actin* gene
1	*Agelaioides badius*	Grayish baywing	Co	MS	+
2	*Agelaioides badius*	Grayish baywing			+
3	*Agelaioides badius*	Grayish baywing			+
4	*Agelaioides badius*	Grayish baywing			+
5	*Agelaioides badius*	Grayish baywing			+
6	*Agelasticus cyanopus*	Unicolored blackbird	NS	MT	+
7	*Agelasticus cyanopus*	Unicolored blackbird			+
8	*Agelasticus cyanopus*	Unicolored blackbird			+
9	*Antilophia galeata*	Helmeted manakin	NS	MT	+
10	*Aramides cajanea*	Gray-necked Wood-Rail	NS	MT	-
11	*Arremon flavirostris*	Saffron-billed sparrow	Po, SA	MT	+
12	*Arremon flavirostris*	Saffron-billed sparrow			+
13	*Arremon flavirostris*	Saffron-billed sparrow			+
14	*Arremon flavirostris*	Saffron-billed sparrow			+
15	*Arremon flavirostris*	Saffron-billed sparrow			+
16	*Arremon flavirostris*	Saffron-billed sparrow			+
17	*Arremon flavirostris*	Saffron-billed sparrow			+
18	*Arundinicola leucocephala*	White-headed marsh tyrant	NS	MT	+
19	*Arundinicola leucocephala*	White-headed marsh tyrant			+
20	*Arundinicola leucocephala*	White-headed marsh tyrant			+
21	*Arundinicola leucocephala*	White-headed marsh tyrant			+
22	*Basileuterus flaveolus*	Flavescent warbler	Po, SA	MT	+
23	*Basileuterus flaveolus*	Flavescent warbler			+
24	*Basileuterus flaveolus*	Flavescent warbler			+
25	*Basileuterus flaveolus*	Flavescent warbler			+
26	*Basileuterus flaveolus*	Flavescent warbler			+
27	*Basileuterus flaveolus*	Flavescent warbler			+
28	*Basileuterus flaveolus*	Flavescent warbler			+
29	*Basileuterus flaveolus*	Flavescent warbler			+
30	*Basileuterus flaveolus*	Flavescent warbler			+
31	*Basileuterus flaveolus*	Flavescent warbler			+
32	*Basileuterus flaveolus*	Flavescent warbler			+
33	*Basileuterus flaveolus*	Flavescent warbler			+
34	*Basileuterus flaveolus*	Flavescent warbler			+
35	*Basileuterus flaveolus*	Flavescent warbler			+

36	*Basileuterus flaveolus*	Flavescent warbler			+
37	*Basileuterus flaveolus*	Flavescent warbler			+
38	*Basileuterus flaveolus*	Flavescent warbler			+
39	*Basileuterus flaveolus*	Flavescent warbler			+
40	*Basileuterus flaveolus*	Flavescent warbler			+
41	*Basileuterus flaveolus*	Flavescent warbler			+
42	*Basileuterus flaveolus*	Flavescent warbler			+
43	*Basileuterus flaveolus*	Flavescent warbler			+
44	*Basileuterus flaveolus*	Flavescent warbler			+
45	*Basileuterus hypoleucus*	White-bellied warbler	SA	MT	+
46	*Basileuterus hypoleucus*	White-bellied warbler			+
47	*Basileuterus hypoleucus*	White-bellied warbler			+
48	*Basileuterus hypoleucus*	White-bellied warbler			+
49	*Cacicus cela*	Yellow-rumped cacique	Po	MT	+
50	*Cacicus cela*	Yellow-rumped cacique			+
51	*Cacicus cela*	Yellow-rumped cacique			+
52	*Cacicus cela*	Yellow-rumped cacique			+
53	*Cacicus cela*	Yellow-rumped cacique			-
54	*Cacicus cela*	Yellow-rumped cacique			+
55	*Cacicus cela*	Yellow-rumped cacique			+
56	*Cacicus cela*	Yellow-rumped cacique			-
57	*Cacicus cela*	Yellow-rumped cacique			+
58	*Cacicus cela*	Yellow-rumped cacique			+
59	*Cacicus cela*	Yellow-rumped cacique			+
60	*Cacicus cela*	Yellow-rumped cacique			+
61	*Cacicus solitarius*	Solitary cacique	NS, Po	MT	+
62	*Cacicus solitarius*	Solitary cacique			+
63	*Campylorhynchus turdinus*	Thrush-like wren	*SA*	MT	+
64	*Campylorhynchus turdinus*	Thrush-like wren			+
65	*Cantorchilus leucotis*	Buff-breasted wren	*NS, Co, SA,*	MT, MS	+
66	*Cantorchilus leucotis*	Buff-breasted wren			+
67	*Cantorchilus leucotis*	Buff-breasted wren			+
68	*Cantorchilus leucotis*	Buff-breasted wren			+
69	*Cantorchilus leucotis*	Buff-breasted wren			+
70	*Cantorchilus leucotis*	Buff-breasted wren			+
71	*Casiornis rufus*	rufous casiornis	*SA*	MT	+
72	*Cercomacra melanaria*	Mato grosso antbird	*Po, NS, SA*	MT	+
73	*Cercomacra melanaria*	Mato grosso antbird			+
74	*Cercomacra melanaria*	Mato grosso antbird			+
75	*Cercomacra melanaria*	Mato grosso antbird			+
76	*Cercomacra melanaria*	Mato grosso antbird			+
77	*Cercomacra melanaria*	Mato grosso antbird			+
78	*Cercomacra melanaria*	Mato grosso antbird			+
79	*Cercomacra melanaria*	Mato grosso antbird			-
80	*Cercomacra melanaria*	Mato grosso antbird			+
81	*Cercomacra melanaria*	Mato grosso antbird			+

82	*Certhiaxis cinnamomeus*	Yellow-chinned spine tail	*Po, NS, SA*	MT	+
83	*Certhiaxis cinnamomeus*	Yellow-chinned spine tail			+
84	*Certhiaxis cinnamomeus*	Yellow-chinned spine tail			+
85	*Certhiaxis cinnamomeus*	Yellow-chinned spine tail			+
86	*Certhiaxis cinnamomeus*	Yellow-chinned spine tail			+
87	*Certhiaxis cinnamomeus*	Yellow-chinned spine tail			+
88	*Certhiaxis cinnamomeus*	Yellow-chinned spine tail			+
89	*Certhiaxis cinnamomeus*	Yellow-chinned spine tail			+
90	*Certhiaxis cinnamomeus*	Yellow-chinned spine tail			+
91	*Certhiaxis cinnamomeus*	Yellow-chinned spine tail			+
92	*Certhiaxis cinnamomeus*	Yellow-chinned spine tail			+
93	*Certhiaxis cinnamomeus*	Yellow-chinned spine tail			+
94	*Certhiaxis cinnamomeus*	Yellow-chinned spine tail			+
95	*Certhiaxis cinnamomeus*	Yellow-chinned spine tail			+
96	*Certhiaxis cinnamomeus*	Yellow-chinned spine tail			+
97	*Certhiaxis cinnamomeus*	Yellow-chinned spine tail			+
98	*Certhiaxis cinnamomeus*	Yellow-chinned spine tail			+
99	*Certhiaxis cinnamomeus*	Yellow-chinned spine tail			+
100	*Cnemotriccus fuscatus*	Fuscous flycatcher	*Po, NS, SA*	MT	+
101	*Cnemotriccus fuscatus*	Fuscous flycatcher			-
102	*Cnemotriccus fuscatus*	Fuscous flycatcher			+
103	*Cnemotriccus fuscatus*	Fuscous flycatcher			+
104	*Cnemotriccus fuscatus*	Fuscous flycatcher			+
105	*Cnemotriccus fuscatus*	Fuscous flycatcher			+
106	*Coccycua minuta*	Little Cuckoo	*SA*	MT	-
107	*Coereba flaveola*	Bananaquit	*Po, SA*	MT	+
108	*Coereba flaveola*	Bananaquit			+
109	*Coereba flaveola*	Bananaquit			+
110	*Coereba flaveola*	Bananaquit			+
111	*Conirostrum speciosum*	Chesnut-vented conebill	*SA*	MT	+
112	*Coryphospingus cucullatus*	Red-crested finch	*SA*	MT	+
113	*Cranioleuca vulpina*	Rusty-backed spinetail	*NS, SA*	MT	+
114	*Cranioleuca vulpina*	Rusty-backed spinetail			+
115	*Cranioleuca vulpina*	Rusty-backed spinetail			+
116	*Cyanocorax chrysops*	Plush-crested jay	*Co*	MS	+
117	*Cyanocorax cyanomelas*	Purplish jay	*SA, NS, Co*	MT, MS	+
118	*Cyanocorax cyanomelas*	Purplish jay			+
119	*Cyanocorax cyanomelas*	Purplish jay			-
120	*Cyanocorax cyanomelas*	Purplish jay			+
121	*Cyclarhis gujanensis*	Rufous-browed peppershrike	*Co*	MS	+
122	*Dendroplex picus*	Straight-billed woodcreeper	*Co*	MS	+
123	*Dendroplex picus*	Straight-billed woodcreeper			+
124	*Dendroplex picus*	Straight-billed woodcreeper			+

125	*Dendroplex picus*	Straight-billed woodcreeper			+
126	*Dendroplex picus*	Straight-billed woodcreeper			+
127	*Dendroplex picus*	Straight-billed woodcreeper			+
128	*Donacobius atricapilla*	Black-capped donacobius			-
129	*Donacobius atricapilla*	Black-capped donacobius			-
130	*Donacobius atricapilla*	Black-capped donacobius			+
131	*Donacobius atricapilla*	Black-capped donacobius			+
132	*Donacobius atricapilla*	Black-capped donacobius			+
133	*Donacobius atricapilla*	Black-capped donacobius			+
134	*Donacobius atricapilla*	Black-capped donacobius			+
135	*Elaenia albiceps*	White-crested elaenia	*Po, NS*	MT	+
136	*Elaenia albiceps*	White-crested elaenia			+
137	*Elaenia spectabilis*	White-crested elaenia	*SA*	MT	+
138	*Eucometis penicillata*	Gray-headed tanager	*SA*	MT	+
139	*Eucometis penicillata*	Gray-headed tanager			+
140	*Eucometis penicillata*	Gray-headed tanager			-
141	*Eucometis penicillata*	Gray-headed tanager			+
142	*Eucometis penicillata*	Gray-headed tanager			+
143	*Eucometis penicillata*	Gray-headed tanager			+
144	*Euscarthmus melorhyphus*	Fulvous-crowned scrub tyrant	*SA*	MT	+
145	*Euscarthmus melorhyphus*	Fulvous-crowned scrub tyrant			+
146	*Euscarthmus melorhyphus*	Fulvous-crowned scrub tyrant			+
147	*Fluvicola albiventer*	Black-backed water tyrant	*NS*	MT	+
148	*Fluvicola albiventer*	Black-backed water tyrant			+
149	*Fluvicola albiventer*	Black-backed water tyrant			+
150	*Fluvicola albiventer*	Black-backed water tyrant			+
151	*Fluvicola albiventer*	Black-backed water tyrant			+
152	*Fluvicola albiventer*	Black-backed water tyrant			+
153	*Fluvicola albiventer*	Black-backed water tyrant			+
154	*Fluvicola albiventer*	Black-backed water tyrant			+
155	*Furnarius leucopus*	Pale-legged hornero	*Po, NS, Co*	MT, MS	+
156	*Furnarius leucopus*	Pale-legged hornero			+
157	*Furnarius leucopus*	Pale-legged hornero			+
158	*Furnarius leucopus*	Pale-legged hornero			+
159	*Furnarius leucopus*	Pale-legged hornero			+
160	*Furnarius leucopus*	Pale-legged hornero			+
161	*Furnarius leucopus*	Pale-legged hornero			+
162	*Furnarius leucopus*	Pale-legged hornero			+
163	*Furnarius leucopus*	Pale-legged hornero			+
164	*Furnarius leucopus*	Pale-legged hornero			+
165	*Furnarius leucopus*	Pale-legged hornero			+

166	*Furnarius leucopus*	Pale-legged hornero			+
167	*Furnarius leucopus*	Pale-legged hornero			+
168	*Furnarius rufus*	Rufous hornero	*NS, SA, Po, Co*	MT, MS	+
169	*Furnarius rufus*	Rufous hornero			+
170	*Furnarius rufus*	Rufous hornero			+
171	*Furnarius rufus*	Rufous hornero			+
172	*Furnarius rufus*	Rufous hornero			+
173	*Furnarius rufus*	Rufous hornero			+
174	*Furnarius rufus*	Rufous hornero			+
175	*Furnarius rufus*	Rufous hornero			+
176	*Furnarius rufus*	Rufous hornero			+
177	*Furnarius rufus*	Rufous hornero			+
178	*Furnarius rufus*	Rufous hornero			+
179	*Furnarius rufus*	Rufous hornero			+
180	*Furnarius rufus*	Rufous hornero			+
181	*Furnarius rufus*	Rufous hornero			+
182	*Furnarius rufus*	Rufous hornero			+
183	*Furnarius rufus*	Rufous hornero			+
184	*Furnarius rufus*	Rufous hornero			+
185	*Furnarius rufus*	Rufous hornero			+
186	*Furnarius rufus*	Rufous hornero			+
187	*Furnarius rufus*	Rufous hornero			+
188	*Hemitriccus margaritaceiventer*	Pearly-vented Tody-tyrant	*Po*	MT	+
189	*Hemitriccus striaticollis*	Stripe-necked Tody-tyrant	*NS, SA*	MT	+
190	*Hemitriccus striaticollis*	Stripe-necked Tody-tyrant			+
191	*Hemitriccus striaticollis*	Stripe-necked Tody-tyrant			+
192	*Hemitriccus striaticollis*	Stripe-necked Tody-tyrant			+
193	*Hemitriccus striaticollis*	Stripe-necked Tody-tyrant			-
194	*Hemitriccus striaticollis*	Stripe-necked Tody-tyrant			+
195	*Herpsilochmus longirostris*	Large-billed antwren	*NS*	MT	+
196	*Hylophilus pectoralis*	Ashy-headed greenlet	*SA*	MT	+
197	*Hylophilus pectoralis*	Ashy-headed greenlet			+
198	*Hylophilus pectoralis*	Ashy-headed greenlet			+
199	*Hypocnemoides maculicauda*	Band-tailed antbird	*SA, NS, Po*	MT	+
200	*Hypocnemoides maculicauda*	Band-tailed antbird			+
201	*Hypocnemoides maculicauda*	Band-tailed antbird			+
202	*Hypocnemoides maculicauda*	Band-tailed antbird			+
203	*Hypocnemoides maculicauda*	Band-tailed antbird			+
204	*Hypocnemoides maculicauda*	Band-tailed antbird			+

205	*Hypocnemoides maculicauda*	Band-tailed antbird			+
206	*Icterus cayanensis*	Epaulet oriole	*SA*<br>*Co*	MT<br>MS	+
207	*Icterus cayanensis*	Epaulet oriole			+
208	*Icterus croconotus*	Orange-backed troupial	*SA*	MT	+
209	*Legatus leucophaius*	Piratic flycatcher	*Po*	MT	+
210	*Legatus leucophaius*	Piratic flycatcher			+
211	*Legatus leucophaius*	Piratic flycatcher			+
212	*Legatus leucophaius*	Piratic flycatcher			+
213	*Lepidocolaptes angustirostris*	Narrow-billed woodcreeper	*Co*	MS	+
214	*Machetornis rixosa*	Cattle tyrant	*NS, Co*	MT, MS	+
215	*Machetornis rixosa*	Cattle tyrant			+
216	*Machetornis rixosa*	Cattle tyrant			+
217	*Machetornis rixosa*	Cattle tyrant			+
218	*Machetornis rixosa*	Cattle tyrant			+
219	*Molothrus oryzivorus*	Giant cowbird	*Co*	MS	+
220	*Myiarchus ferox*	Short-crested flycatcher	*Po, SA, NS*<br>*SA*<br>*Po, NS, Co*<br>*NA, SA, Po, Co*<br>*Po, SA, NS*<br>*SA*	MT<br>MT<br>MT, MS<br>MT, MS<br>MT<br>MT	+
221	*Myiarchus ferox*	Short-crested flycatcher			+
222	*Myiarchus ferox*	Short-crested flycatcher			+
223	*Myiarchus ferox*	Short-crested flycatcher			+
224	*Myiarchus ferox*	Short-crested flycatcher			+
225	*Myiarchus ferox*	Short-crested flycatcher			+
226	*Myiophobus fasciatus*	Bran-colored flycatcher	*Po, NS, Co*<br>*NA, SA, Po, Co*<br>*Po, SA, NS*	MT, MS<br>MT, MS<br>MT	+
227	*Myiophobus fasciatus*	Bran-colored flycatcher			+
228	*Myiophobus fasciatus*	Bran-colored flycatcher			+
229	*Myiozetetes cayanensis*	Rusty-margined flycatchter	*SA*<br>*Po, NS, Co*<br>*NA, SA, Po, Co*<br>*Po, SA, NS*<br>*SA*	MT<br>MT, MS<br>MT, MS<br>MT<br>MT	+
230	*Myiozetetes cayanensis*	Rusty-margined flycatchter			+
231	*Myiozetetes cayanensis*	Rusty-margined flycatchter			+
232	*Myiozetetes cayanensis*	Rusty-margined flycatchter			+
233	*Myiozetetes cayanensis*	Rusty-margined flycatchter			+
234	*Paroaria capitata*	Yellow-billed cardinal	*Po, NS, Co*	MT, MS	+
235	*Paroaria capitata*	Yellow-billed cardinal			+
236	*Paroaria capitata*	Yellow-billed cardinal			+
237	*Paroaria capitata*	Yellow-billed cardinal			+
238	*Paroaria capitata*	Yellow-billed cardinal			+
239	*Paroaria capitata*	Yellow-billed cardinal			+
240	*Paroaria capitata*	Yellow-billed cardinal			+
241	*Paroaria capitata*	Yellow-billed cardinal			+

242	*Paroaria capitata*	Yellow-billed cardinal			+
243	*Paroaria capitata*	Yellow-billed cardinal			+
244	*Paroaria capitata*	Yellow-billed cardinal			+
245	*Paroaria capitata*	Yellow-billed cardinal			+
246	*Paroaria capitata*	Yellow-billed cardinal			+
247	*Paroaria capitata*	Yellow-billed cardinal			+
248	*Paroaria capitata*	Yellow-billed cardinal			+
249	*Paroaria capitata*	Yellow-billed cardinal			+
250	*Paroaria capitata*	Yellow-billed cardinal			+
251	*Paroaria capitata*	Yellow-billed cardinal			+
252	*Paroaria capitata*	Yellow-billed cardinal			+
253	*Paroaria capitata*	Yellow-billed cardinal			+
254	*Paroaria capitata*	Yellow-billed cardinal			+
255	*Paroaria capitata*	Yellow-billed cardinal			+
256	*Paroaria capitata*	Yellow-billed cardinal			+
257	*Paroaria capitata*	Yellow-billed cardinal			+
258	*Paroaria capitata*	Yellow-billed cardinal			+
259	*Paroaria capitata*	Yellow-billed cardinal			+
260	*Paroaria capitata*	Yellow-billed cardinal			+
261	*Paroaria capitata*	Yellow-billed cardinal			+
262	*Paroaria capitata*	Yellow-billed cardinal			+
263	*Paroaria capitata*	Yellow-billed cardinal			+
264	*Paroaria capitata*	Yellow-billed cardinal			+
265	*Paroaria capitata*	Yellow-billed cardinal			+
266	*Paroaria capitata*	Yellow-billed cardinal			+
267	*Paroaria capitata*	Yellow-billed cardinal			+
268	*Paroaria capitata*	Yellow-billed cardinal			+
269	*Paroaria capitata*	Yellow-billed cardinal			+
270	*Picumnus albosquamatus*	White-wedged Piculet	*NS, SA*<br>*Po, SA*<br>*NS, SA*<br>*Po, SA*	MT<br>MT<br>MT<br>MT	-
271	*Picumnus albosquamatus*	White-wedged Piculet			-
272	*Picumnus albosquamatus*	White-wedged Piculet			-
273	*Picumnus albosquamatus*	White-wedged Piculet			-
274	*Pipra fasciicauda*	Band-tailed manakin	*NS, SA*	MT	+
275	*Pipra fasciicauda*	Band-tailed manakin			+
276	*Pipra fasciicauda*	Band-tailed manakin			+
277	*Pipra fasciicauda*	Band-tailed manakin			+
278	*Pipra fasciicauda*	Band-tailed manakin			+
279	*Pipra fasciicauda*	Band-tailed manakin			+
280	*Pipra fasciicauda*	Band-tailed manakin			+
281	*Pitangus sulphuratus*	Great kiskadee	*SA, NS,*<br>*Po, Co*	MT,<br>MS	+
282	*Pitangus sulphuratus*	Great kiskadee			+
283	*Pitangus sulphuratus*	Great kiskadee			+
284	*Pitangus sulphuratus*	Great kiskadee			+
285	*Pitangus sulphuratus*	Great kiskadee			+
286	*Pitangus sulphuratus*	Great kiskadee			+
287	*Pitangus sulphuratus*	Great kiskadee			+

288	*Pitangus sulphuratus*	Great kiskadee			+
289	*Pitangus sulphuratus*	Great kiskadee			+
290	*Pitangus sulphuratus*	Great kiskadee			+
291	*Pitangus sulphuratus*	Great kiskadee			+
292	*Pitangus sulphuratus*	Great kiskadee			+
293	*Pitangus sulphuratus*	Great kiskadee			+
294	*Pitangus sulphuratus*	Great kiskadee			+
295	*Pitangus sulphuratus*	Great kiskadee			+
296	*Pitangus sulphuratus*	Great kiskadee			+
297	*Pitangus sulphuratus*	Great kiskadee			+
298	*Pitangus sulphuratus*	Great kiskadee			+
299	*Pitangus sulphuratus*	Great kiskadee			+
300	*Pitangus sulphuratus*	Great kiskadee			+
301	*Pitangus sulphuratus*	Great kiskadee			+
302	*Pitangus sulphuratus*	Great kiskadee			+
303	*Pitangus sulphuratus*	Great kiskadee			+
304	*Pitangus sulphuratus*	Great kiskadee			+
305	*Pitangus sulphuratus*	Great kiskadee			+
306	*Poecilotriccus latirostris*	Rusty-fronted Tody-Flycatcher	*Po*	MT	+
307	*Poecilotriccus latirostris*	Rusty-fronted Tody-Flycatcher			+
308	*Poecilotriccus latirostris*	Rusty-fronted Tody-Flycatcher			+
309	*Poecilotriccus latirostris*	Rusty-fronted Tody-Flycatcher			+
310	*Poecilotriccus latirostris*	Rusty-fronted Tody-Flycatcher			+
311	*Poecilotriccus latirostris*	Rusty-fronted Tody-Flycatcher			+
312	*Poecilotriccus latirostris*	Rusty-fronted Tody-Flycatcher			+
313	*Poecilotriccus latirostris*	Rusty-fronted Tody-Flycatcher			+
314	*Progne tapera*	Brown-chested martin	*NS*	MT	+
315	*Pseudoseisura unirufa*	Grey-crested cacholote	*NS, SA, Po Co*	MT, MS	+
316	*Pseudoseisura unirufa*	Grey-crested cacholote			+
317	*Pseudoseisura unirufa*	Grey-crested cacholote			+
318	*Pseudoseisura unirufa*	Grey-crested cacholote			+
319	*Pseudoseisura unirufa*	Grey-crested cacholote			+
320	*Pseudoseisura unirufa*	Grey-crested cacholote			+
321	*Pseudoseisura unirufa*	Grey-crested cacholote			+
322	*Pseudoseisura unirufa*	Grey-crested cacholote			+
323	*Ramphocelus carbo*	Silver-beaked tanager	*NS, SA, Po, Co*	MT, MS	+
324	*Ramphocelus carbo*	Silver-beaked tanager			+
325	*Ramphocelus carbo*	Silver-beaked tanager			+
326	*Ramphocelus carbo*	Silver-beaked tanager			+

327	*Ramphocelus carbo*	Silver-beaked tanager			+
328	*Ramphocelus carbo*	Silver-beaked tanager			+
329	*Ramphocelus carbo*	Silver-beaked tanager			+
330	*Ramphocelus carbo*	Silver-beaked tanager			-
331	*Ramphocelus carbo*	Silver-beaked tanager			+
332	*Ramphocelus carbo*	Silver-beaked tanager			+
333	*Ramphocelus carbo*	Silver-beaked tanager			+
334	*Ramphocelus carbo*	Silver-beaked tanager			+
335	*Ramphocelus carbo*	Silver-beaked tanager			+
336	*Ramphocelus carbo*	Silver-beaked tanager			+
337	*Ramphocelus carbo*	Silver-beaked tanager			+
338	*Ramphocelus carbo*	Silver-beaked tanager			+
339	*Ramphocelus carbo*	Silver-beaked tanager			+
340	*Ramphocelus carbo*	Silver-beaked tanager			+
341	*Ramphocelus carbo*	Silver-beaked tanager			+
342	*Ramphocelus carbo*	Silver-beaked tanager			+
343	*Ramphocelus carbo*	Silver-beaked tanager			+
344	*Ramphocelus carbo*	Silver-beaked tanager			+
345	*Ramphocelus carbo*	Silver-beaked tanager			+
346	*Ramphocelus carbo*	Silver-beaked tanager			+
347	*Ramphocelus carbo*	Silver-beaked tanager			+
348	*Ramphocelus carbo*	Silver-beaked tanager			+
349	*Ramphocelus carbo*	Silver-beaked tanager			+
350	*Ramphocelus carbo*	Silver-beaked tanager			+
351	*Ramphocelus carbo*	Silver-beaked tanager			+
352	*Ramphocelus carbo*	Silver-beaked tanager			+
353	*Ramphocelus carbo*	Silver-beaked tanager			+
354	*Ramphocelus carbo*	Silver-beaked tanager			+
355	*Ramphocelus carbo*	Silver-beaked tanager			+
356	*Ramphocelus carbo*	Silver-beaked tanager			+
357	*Ramphocelus carbo*	Silver-beaked tanager			+
358	*Ramphocelus carbo*	Silver-beaked tanager			+
359	*Ramphocelus carbo*	Silver-beaked tanager			+
360	*Ramphocelus carbo*	Silver-beaked tanager			+
361	*Ramphocelus carbo*	Silver-beaked tanager			+
362	*Ramphocelus carbo*	Silver-beaked tanager			+
363	*Ramphocelus carbo*	Silver-beaked tanager			+
364	*Ramphocelus carbo*	Silver-beaked tanager			+
365	*Ramphocelus carbo*	Silver-beaked tanager			+
366	*Ramphocelus carbo*	Silver-beaked tanager			+
367	*Ramphocelus carbo*	Silver-beaked tanager			+
368	*Ramphocelus carbo*	Silver-beaked tanager			+
369	*Ramphocelus carbo*	Silver-beaked tanager			+
370	*Ramphocelus carbo*	Silver-beaked tanager			+
371	*Ramphocelus carbo*	Silver-beaked tanager			+
372	*Ramphocelus carbo*	Silver-beaked tanager			+

373	*Ramphocelus carbo*	Silver-beaked tanager			+
374	*Ramphocelus carbo*	Silver-beaked tanager			+
375	*Ramphocelus carbo*	Silver-beaked tanager			+
376	*Ramphocelus carbo*	Silver-beaked tanager			+
377	*Ramphocelus carbo*	Silver-beaked tanager			+
378	*Ramphocelus carbo*	Silver-beaked tanager			+
379	*Ramphocelus carbo*	Silver-beaked tanager			+
380	*Ramphocelus carbo*	Silver-beaked tanager			+
381	*Ramphocelus carbo*	Silver-beaked tanager			+
382	*Ramphocelus carbo*	Silver-beaked tanager			+
383	*Ramphocelus carbo*	Silver-beaked tanager			+
384	*Ramphocelus carbo*	Silver-beaked tanager			+
385	*Ramphocelus carbo*	Silver-beaked tanager			+
386	*Ramphocelus carbo*	Silver-beaked tanager			+
387	*Ramphocelus carbo*	Silver-beaked tanager			+
388	*Ramphocelus carbo*	Silver-beaked tanager			+
389	*Ramphocelus carbo*	Silver-beaked tanager			+
390	*Ramphocelus carbo*	Silver-beaked tanager			+
391	*Ramphocelus carbo*	Silver-beaked tanager			+
392	*Ramphocelus carbo*	Silver-beaked tanager			+
393	*Ramphocelus carbo*	Silver-beaked tanager			+
394	*Ramphocelus carbo*	Silver-beaked tanager			+
395	*Ramphocelus carbo*	Silver-beaked tanager			+
396	*Ramphocelus carbo*	Silver-beaked tanager			+
397	*Ramphocelus carbo*	Silver-beaked tanager			+
398	*Ramphocelus carbo*	Silver-beaked tanager			+
399	*Ramphocelus carbo*	Silver-beaked tanager			+
400	*Ramphocelus carbo*	Silver-beaked tanager			+
401	*Ramphocelus carbo*	Silver-beaked tanager			+
402	*Ramphocelus carbo*	Silver-beaked tanager			+
403	*Ramphocelus carbo*	Silver-beaked tanager			+
404	*Ramphocelus carbo*	Silver-beaked tanager			+
405	*Ramphocelus carbo*	Silver-beaked tanager			+
406	*Ramphocelus carbo*	Silver-beaked tanager			+
407	*Ramphocelus carbo*	Silver-beaked tanager			+
408	*Ramphocelus carbo*	Silver-beaked tanager			+
409	*Ramphocelus carbo*	Silver-beaked tanager			+
410	*Ramphocelus carbo*	Silver-beaked tanager			+
411	*Ramphocelus carbo*	Silver-beaked tanager			+
412	*Ramphocelus carbo*	Silver-beaked tanager			+
413	*Ramphocelus carbo*	Silver-beaked tanager			+
414	*Ramphocelus carbo*	Silver-beaked tanager			+
415	*Ramphocelus carbo*	Silver-beaked tanager			+
416	*Ramphocelus carbo*	Silver-beaked tanager			+
417	*Ramphocelus carbo*	Silver-beaked tanager			+
418	*Ramphocelus carbo*	Silver-beaked tanager			+

419	*Ramphocelus carbo*	Silver-beaked tanager			+
420	*Ramphocelus carbo*	Silver-beaked tanager			+
421	*Ramphocelus carbo*	Silver-beaked tanager			+
422	*Ramphocelus carbo*	Silver-beaked tanager			+
423	*Ramphocelus carbo*	Silver-beaked tanager			+
424	*Ramphocelus carbo*	Silver-beaked tanager			+
425	*Saltator coerulescens*	Bluish-gray saltator	*NS, SA,*<br>*Po, Co*	MT,<br>MS	+
426	*Saltator coerulescens*	Bluish-gray saltator			+
427	*Saltator coerulescens*	Bluish-gray saltator			+
428	*Saltator coerulescens*	Bluish-gray saltator			+
429	*Saltator coerulescens*	Bluish-gray saltator			+
430	*Saltator coerulescens*	Bluish-gray saltator			+
431	*Saltator coerulescens*	Bluish-gray saltator			+
432	*Saltator coerulescens*	Bluish-gray saltator			+
433	*Saltator coerulescens*	Bluish-gray saltator			+
434	*Saltator coerulescens*	Bluish-gray saltator			+
435	*Saltator coerulescens*	Bluish-gray saltator			+
436	*Saltator coerulescens*	Bluish-gray saltator			+
437	*Saltator coerulescens*	Bluish-gray saltator			+
438	*Saltator coerulescens*	Bluish-gray saltator			+
439	*Saltator coerulescens*	Bluish-gray saltator			+
440	*Saltator coerulescens*	Bluish-gray saltator			+
441	*Saltator coerulescens*	Bluish-gray saltator			+
442	*Saltator coerulescens*	Bluish-gray saltator			+
443	*Sicalis flaveola*	Saffron finch	*NS*<br>*SA, Po*	MT<br>MT	+
444	*Sicalis flaveola*	Saffron finch			+
445	*Sporophila angolensis*	Chesnut-belliend finch	*SA*<br>*NS*<br>*SA, Po*<br>*SA*<br>*NS*<br>*SA, Po*<br>*SA*<br>*NS*<br>*SA, Po*	MT<br>MT<br>MT<br>MT<br>MT<br>MT<br>MT<br>MT<br>MT	+
446	*Sporophila angolensis*	Chesnut-belliend finch			+
447	*Sporophila angolensis*	Chesnut-belliend finch			+
448	*Sporophila angolensis*	Chesnut-belliend finch			+
449	*Sporophila angolensis*	Chesnut-belliend finch			+
450	*Sporophila angolensis*	Chesnut-belliend finch			+
451	*Sporophila angolensis*	Chesnut-belliend finch			+
452	*Sporophila angolensis*	Chesnut-belliend finch			+
453	*Sporophila angolensis*	Chesnut-belliend finch			+
454	*Sporophila caerulescens*	Double-collared seedeater	*SA*	MT	+
455	*Sporophila collaris*	Rusty-collared seedeater	*NS, Po*<br>*SA*<br>*NS, Po*<br>*SA*<br>*NS, Po*<br>*SA*<br>*NS, Po*<br>*SA*<br>*NS, Po*<br>*SA*<br>*NS, Po*	MT<br>MT<br>MT<br>MT<br>MT<br>MT<br>MT<br>MT<br>MT<br>MT<br>MT	+
456	*Sporophila collaris*	Rusty-collared seedeater			+
457	*Sporophila collaris*	Rusty-collared seedeater			+
458	*Sporophila collaris*	Rusty-collared seedeater			+
459	*Sporophila collaris*	Rusty-collared seedeater			+
460	*Sporophila collaris*	Rusty-collared seedeater			+
461	*Sporophila collaris*	Rusty-collared seedeater			+
462	*Sporophila collaris*	Rusty-collared seedeater			+
463	*Sporophila collaris*	Rusty-collared seedeater			+
464	*Sporophila collaris*	Rusty-collared seedeater			+

465	*Sporophila collaris*	Rusty-collared seedeater			+
466	*Sporophila lineola*	Lined seedeater	*SA*	MT	+
467	*Stelgidopteryx ruficollis*	Southern roughed-wing swallow	*SA*	MT	+
468	*Stelgidopteryx ruficollis*	Southern roughed-wing swallow			+
469	*Stelgidopteryx ruficollis*	Southern roughed-wing swallow			+
470	*Synallaxis albilora*	White-lored spinetail	*SA, NS, Po*	MT	+
471	*Synallaxis albilora*	White-lored spinetail			+
472	*Synallaxis albilora*	White-lored spinetail			+
473	*Synallaxis albilora*	White-lored spinetail			+
474	*Synallaxis albilora*	White-lored spinetail			+
475	*Synallaxis albilora*	White-lored spinetail			+
476	*Synallaxis albilora*	White-lored spinetail			+
477	*Synallaxis albilora*	White-lored spinetail			+
478	*Synallaxis albilora*	White-lored spinetail			+
479	*Synallaxis albilora*	White-lored spinetail			+
480	*Taraba major*	Great antshrike	*NS, SA, Po*	MT	+
481	*Taraba major*	Great antshrike			+
482	*Taraba major*	Great antshrike			+
483	*Taraba major*	Great antshrike			+
484	*Thraupis palmarum*	Palm tanager	*NS, Co*	MT, MS	+
485	*Thraupis palmarum*	Palm tanager			-
486	*Thraupis sayaca*	Sayaca tanager	*NS, Co*	MT, MS	+
487	*Thraupis sayaca*	Sayaca tanager			+
488	*Thraupis sayaca*	Sayaca tanager			+
489	*Thryothorus genibarbis*	Moustached wren	*SA*	MT	+
490	*Todirostrum cinereum*	Common-Tody flycatcher	*NS*	MT	+
491	*Todirostrum cinereum*	Common-Tody flycatcher			+
492	*Turdus amaurochalinus*	Creamy-bellied thrush	*SA*	MT	+
493	*Turdus amaurochalinus*	Creamy-bellied thrush			+
494	*Turdus amaurochalinus*	Creamy-bellied thrush			+
495	*Turdus hauxwelli*	Hauwell's thrush	*SA*	MT	+
496	*Turdus leucomelas*	Pale-breasted thrush	*SA, Po*	MT	+
497	*Turdus leucomelas*	Pale-breasted thrush			+
498	*Turdus leucomelas*	Pale-breasted thrush			+
499	*Turdus leucomelas*	Pale-breasted thrush			+
500	*Turdus leucomelas*	Pale-breasted thrush			+
501	*Turdus leucomelas*	Pale-breasted thrush			+
502	*Turdus leucomelas*	Pale-breasted thrush			+
503	*Turdus leucomelas*	Pale-breasted thrush			+
504	*Turdus rufiventris*	Rufous-bellied thrush	*SA, NS, Co*	MT, MS	+
505	*Turdus rufiventris*	Rufous-bellied thrush			+
506	*Turdus rufiventris*	Rufous-bellied thrush			+
507	*Turdus rufiventris*	Rufous-bellied thrush			+

508	*Turdus rufiventris*	Rufous-bellied thrush			+
509	*Turdus rufiventris*	Rufous-bellied thrush			+
510	*Turdus rufiventris*	Rufous-bellied thrush			+
511	*Tyrannus melancholicus*	Tropical kingbird	*NS, SA, Co*	MT, MS	+
512	*Tyrannus melancholicus*	Tropical kingbird			+
513	*Tyrannus melancholicus*	Tropical kingbird			+
514	*Vireo olivaceus*	red-eyed vireo	*NS*	MT	+
515	*Volatinia jacarina*	Blue-black grassquit	*NS, SA*	MT	+
516	*Volatinia jacarina*	Blue-black grassquit			+
517	*Volatinia jacarina*	Blue-black grassquit			+
