# Supplementary material for: Molecular survey of Hepatozoon spp., piroplasmids, and onchocercids in wild birds from the Brazilian Pantanal
Source: Rev Bras Parasitol Vet. 2025 Nov 17;34(4):e010725. doi: 10.1590/S1984-29612025065 (PMC12643243; doi:10.1590/S1984-29612025065)
Supplement: Supplementary Material Table SM2 [file rbpv-34-4-e010725-suppl02.pdf]

Supplementary Material Table SM2. Description of primers and target genes used in the on conventional PCR assays to detect piroplasmids, *Hepatozoon* spp., and filariids in avian samples from the Brazilian Pantanal.

Agents	Sequences (5'-3')	Length (bp)	Target Gene	Reference
Babesia spp.	BTF1 (external) GGCTCATTACAACAGTTATAG	900	18S rRNA	(Jefferies et al., 2007)
	BTR1 (external) CCCAAAGACTTTGATTTCTCTC			
	BTF2 (internal) CCGTGCTAATTGTAGGGCTAATAC	830		
	BTR2 (internal) GGACTACGACGGTATCTGATCG			
Hepatozoon spp.	HEMO1 TATTGGTTTTAAGAACTAATTTTATGATTG	900	18S rRNA	(Perkins & Keller, 2001)
	HEMO2 CTTCTCCTTCCTTTAAGTGATAAGGTTCAC			
Hepatozoon spp.	HepF300 GTTTCTGACCTATCAGCTTTCGACG	600	18S rRNA	(Ujvari et al., 2004)
	Hep900 CAAATCTAAGAATTTCACCTCTGAC			
Filariids	NTF-coxF TGATTGGTGGTTTTGGTAA	650	*cox-1*	(Casiraghi et al., 2001)
	NTR-coxR ATAAGTACGAGTATCAATATC			
Filariids	988F CTCAAAGATTAAGCCATGC	998	12S rRNA	(Hayashi et al., 2024)
	1912R TTTACGGTCAGAACTAGGG			
Filariids	Nematode 1 GCGGAGGAAAAGAAACTAA	855	28S rRNA	(Hayashi et al., 2024)
	Nematode 2 ATCCGTGTTTCAAGACGGG			

References

Casiraghi M, Anderson TJC, Bandi C, Bazzocchi C, Genchi C. A phylogenetic analysis of filarial nematodes: Comparison with the phylogeny of *Wolbachia* endosymbionts. *Parasitology* 2001; 122(1): 93–103. https://doi.org/10.1017/S0031182000007149

Hayashi N, Hosokawa K, Yamamoto Y, Kodama S, Kurokawa A, Nakao R, et al. A filarial parasite potentially associated with the health burden on domestic chickens in Japan. *Sci Rep* 2024; 14(1): 6316. https://doi.org/10.1038/s41598-024-55284-2

Jefferies R, Ryan UM, Irwin PJ. PCR–RFLP for the detection and differentiation of the canine piroplasm species and its use with filter paper-based technologies. *Vet Parasitol* 2007; 144(1–2): 20–27. https://doi.org/10.1016/j.vetpar.2006.09.022

Perkins SL, Keller K. Phylogeny of nuclear small subunit rRNA genes of hemogregarines amplified with specific primers. *J Parasitol* 2001; 87(4): 870–876. https://doi.org/10.1645/0022-3395(2001)087[0870:PONSSR]2.0.CO;2

Ujvari B, Madsen T, Olsson M. High Prevalence of *Hepatozoon* spp. (Apicomplexa, Hepatozoidae) Infection in Water Pythons (*Liasis fuscus*) From Tropical Australia. *J Parasitol* 2004; 90(3): 670–672. https://doi.org/10.1645/GE-204R
